# Timing-dependent skin toxicity phenotypes during enfortumab vedotin plus pembrolizumab therapy for advanced urothelial carcinoma

**DOI:** 10.1007/s10147-026-03148-2

**Published:** 2026-07-27

**Authors:** Mamoru Hashimoto, Lan Inoki, Yutaka Yamamoto, Wataru Fukuokaya, Takafumi Yanagisawa, Keiichiro Mori, Ryoichi Maenosono, Yuki Yoshikawa, Takuya Tsujino, Masanobu Saruta, Takuhisa Nukaya, Yuya Oishi, Keita Tamura, Seitetsu Sugiyama, Hirofumi Morinaka, Kazumasa Komura, Kiyoshi Takahara, Teruo Inamoto, Kazutoshi Fujita

**Affiliations:** 1 Department of Urology, Faculty of Medicine, Kindai University, Osaka, Japan; 2 Department of Urology, The Jikei University School of Medicine, Tokyo, Japan; 3 Department of Urology, Osaka Medical and Pharmaceutical University, Osaka, Japan; 4 Department of Urology, Fujita Health University School of Medicine, Aichi, Japan; 5 Department of Urology, Hamamatsu University School of Medicine, Hamamatsu, Japan; 6 Department of Urology, Kawasaki Medical School, Okayama, Japan; 7 Department of Urology, Kindai University Hospital, 1-14-1 Miharadai, Minami-ku, Sakai, Osaka, 590-0197 Japan

**Keywords:** Urothelial carcinoma, Enfortumab vedotin, Pembrolizumab, Skin toxicity, Adverse events, Prognosis

## Abstract

**Background:**

Enfortumab vedotin plus pembrolizumab (EV/Pem) has improved the survival of patients with advanced urothelial carcinoma; however, many patients suffer treatment-related adverse events, particularly skin toxicity. This study aimed to clarify the clinical impact of skin-toxicity timing on treatment outcomes.

**Methods:**

We retrospectively analyzed patients with advanced urothelial carcinoma treated with EV/Pem. Patients were categorized into three groups according to skin-toxicity timing: no rash, early-onset rash (< 1 month), and late-onset rash (≥ 1 month). Baseline clinical and laboratory characteristics were compared among groups and incorporated into multivariable Cox proportional hazards analyses. Kaplan-Meier analyses with pairwise log-rank tests were performed to evaluate progression-free survival (PFS) and overall survival (OS). The 1-month cutoff was selected for clinical interpretability.

**Results:**

The three-group analysis included 210 patients: no rash (*n* = 87), early-onset rash (*n* = 84), and late-onset rash (*n* = 39). Late-onset rash was associated with significantly prolonged PFS and OS in conventional analyses. In multivariable analysis, late-onset rash was significantly associated with prolonged PFS (HR 0.34, 95% CI 0.15–0.74, *p* = 0.007) and OS (HR 0.25, 95% CI 0.07–0.81, *p* = 0.02). Because late-onset rash could only be assigned after continued observation, these associations should be interpreted as exploratory and potentially affected by immortal time bias. In the 3-month landmark analysis, PFS remained significantly longer in the late-onset than early-onset rash group, whereas OS did not differ significantly.

**Conclusions:**

Skin toxicity during EV/Pem therapy appears to have timing-dependent prognostic implications. Late-onset rash showed favorable outcomes in conventional analyses, but these findings should be considered hypothesis-generating.

## Introduction

 Enfortumab vedotin plus pembrolizumab (EV/Pem) has become a key first-line treatment for advanced urothelial carcinoma (UC) after the EV-302 trial demonstrated significant improvements in progression-free survival (PFS) and overall survival (OS) compared with platinum-based chemotherapy [1]. EV is an antibody-drug conjugate directed against Nectin-4, a cell adhesion molecule highly expressed in UC. After binding to Nectin-4, EV is internalized and releases monomethyl auristatin E, a microtubule-disrupting cytotoxic payload [2]. Previous UC studies, including analyses of molecular profiles, biomarkers, and real-world EV outcomes, have emphasized the heterogeneity of advanced UC and the need for clinically useful response or toxicity markers [3–10]. However, studies directly addressing EV/Pem-associated skin toxicity have mainly focused on incidence, management, or general clinical implications rather than the prognostic impact of rash timing [11–13]. Therefore, the prognostic significance of the timing of EV/Pem-associated skin toxicity remains unclear.

Despite its efficacy, EV/Pem is associated with a characteristic toxicity profile, including skin toxicity, peripheral neuropathy, hyperglycemia, pneumonitis, and immune-related adverse events. Dermatologic toxicity is particularly important because Nectin-4 is expressed not only in UC cells but also in normal epithelial tissues, including the epidermis and sweat glands [2, 11]. Mechanistically, EV-associated skin toxicity is presumed to result from MMAE delivery into Nectin-4-expressing normal epithelial structures, with additional contributions from immune activation when combined with pembrolizumab [11].

Treatment-related skin toxicity is not necessarily a uniform phenomenon. In several anticancer treatment contexts, including EGFR-targeted therapies, skin toxicity has been associated with clinical efficacy, and the timing of cutaneous immune-related adverse events has also been linked to survival outcomes in patients receiving immune checkpoint inhibitors [14–16]. Therefore, the timing of skin toxicity may provide clinically relevant information beyond the mere presence or absence of rash. We investigated the association between skin-toxicity timing and treatment outcomes in patients treated with EV/Pem.

## Methods

### Patients and study design

We retrospectively reviewed patients with advanced UC treated with EV/Pem at multiple Japanese institutions participating in the ULTRA-J collaborative group, including 6 university hospitals and 15 affiliated hospitals. Skin toxicity was defined as treatment-related dermatologic adverse events of any grade according to the Common Terminology Criteria for Adverse Events (CTCAE), version 5.0. Patients were grouped according to skin-toxicity status and timing as follows: no rash, early-onset rash (< 1 month), and late-onset rash (≥ 1 month), based on the timing of rash onset after EV/Pem initiation. Patients with skin toxicity but unavailable timing information were excluded from the three-group timing analysis. The 1-month cutoff was selected for clinical interpretability because events occurring during the first month often influence early dose interruption or reduction, whereas events after 1 month may reflect toxicity emerging after sustained treatment exposure. This cutoff was not intended to represent a validated biological threshold.

This retrospective multicenter study was carried out in accordance with the Declaration of Helsinki and its subsequent amendments. Approval was obtained from the institutional review board of Kindai University Hospital (Approval No. R02-155), and written informed consent was waived due to the retrospective study design.

### Data collection

Baseline clinical variables, metastatic sites, laboratory data, treatment exposure, adverse event profiles, PFS, and OS were extracted. Inflammatory and epithelial-related biomarkers, including KL-6, and neutrophil-to-lymphocyte ratio (NLR) were extracted or calculated using the quality-controlled dataset.

### Statistical analysis

Overall three-group comparisons were performed using the Kruskal-Wallis test for continuous variables and Fisher’s exact test for categorical variables. In addition, exploratory pairwise comparisons between groups were performed using the Mann-Whitney U test for continuous variables and Fisher’s exact test for categorical variables; Holm adjustment was applied for pairwise comparisons.

Survival curves were estimated using the Kaplan-Meier method. Overall survival comparisons were performed using a global log-rank test, followed by exploratory pairwise log-rank analyses. Holm adjustment was applied for pairwise survival comparisons. Cox proportional hazards models were used for univariate and multivariable analyses of OS and PFS. All statistical analyses were executed using EZR as reported previously (Saitama Medical Center, Jichi Medical University, Saitama, Japan), which is a graphical user interface for R (The R Foundation for Statistical Computing, Vienna, Austria) [17]. Variables with *p* < 0.05 in univariate analysis were candidates for the multivariable models, and age was included as a clinically relevant covariate irrespective of its univariate p value. To address immortal time bias, a 3-month landmark analysis was additionally performed by excluding patients who experienced progression or death within 3 months after EV/Pem initiation or who had no follow-up beyond the 3-month landmark. PFS and OS were recalculated from the 3-month landmark. The original skin-toxicity timing groups were used in the landmark cohort.

## Results

A total of 246 patient records were screened. After excluding patients with inadequate follow-up, no radiographic evaluation, or unavailable timing of skin toxicity, 210 patients were included in the three-group timing analysis. The three-group analysis included 210 patients: no rash (*n* = 87), early-onset rash (*n* = 84), and late-onset rash (*n* = 39). Baseline clinical and laboratory characteristics and disease status are shown in Table 1, and treatment-related outcomes are shown in Table 2. In the pairwise comparisons, KL-6 was significantly lower in the late-onset rash group than in the early-onset rash group after Holm adjustment (Holm-adjusted *p* = 0.02). KL-6 did not differ significantly between the late-onset rash and no-rash groups after Holm adjustment (Holm-adjusted *p* = 0.19). NLR did not clearly separate the groups.

**Table 1:** Baseline variables according to skin-rash timing

Variable	No rash	Early-onset rash (< 1 month)	Late-onset rash ( > = 1 month)	*p* overall	*p* Early vs. No rash	*p* Late vs. No rash	*p* Late vs. Early
Age, years	77 [70–81]	75 [69–79]	75 [66–79.5]	0.52	0.83	0.87	0.88
Female sex	27/87 (31.0%)	24/84 (28.6%)	9/39 (23.1%)	0.69	1.00	1.00	1.00
ECOG PS > = 1	24/87 (27.6%)	27/84 (32.1%)	10/39 (25.6%)	0.75	1.00	1.00	1.00
BMI, kg/m2	21.1 [18.95–23.65]	21.9 [19.9–24.42]	21 [18.75–23.5]	0.11	0.23	0.70	0.23
Albumin, g/dL	3.6 [3.25–3.9]	3.7 [3.3–4]	3.65 [3.3–3.88]	0.73	1.00	1.00	1.00
eGFR, mL/min/1.73m2	47.3 [36–61.08]	43.45 [35–56.65]	46.5 [37.07–56.38]	0.63	1.00	1.00	1.00
CRP, mg/dL	0.89 [0.22–3.4]	0.54 [0.16–2.54]	0.99 [0.29–3.33]	0.20	0.37	0.79	0.37
KL-6, U/mL	269.5 [202.5–394.5]	303 [224–400]	232.5 [174.5–295.25]	**0.03**	0.19	0.19	**0.02**
Neutrophil count, /µL	4751.5 [3700–6675]	4800 [3700–6635]	4316 [3400–7793]	0.94	1.00	1.00	1.00
Lymphocyte count, /uL	1200 [910.25–1600]	1400 [935–1716.5]	1015.5 [685–1867.5]	0.27	0.59	0.59	0.45
NLR	3.96 [2.47–5.58]	3.74 [2.63–5.21]	4.24 [2.29–6.38]	0.78	1.00	1.00	1.00
Liver metastasis	10/86 (11.6%)	10/84 (11.9%)	1/39 (2.6%)	0.24	1.000	0.51	0.51
Lung metastasis	22/86 (25.6%)	25/83 (30.1%)	10/39 (25.6%)	0.77	1.000	1.000	1.000
Bone metastasis	16/86 (18.6%)	11/84 (13.1%)	4/39 (10.3%)	0.44	0.90	0.90	0.90

Data are presented as median [interquartile range] or number/total number (percentage). Pairwise p values were adjusted using the Holm method. Bold values indicate statistical significance (p < 0.05).

BMI, body mass index; eGFR, estimated glomerular filtration rate; CRP, C-reactive protein; KL-6, Krebs von den Lungen-6; NLR, neutrophil-to-lymphocyte ratio; ECOG, Eastern Cooperative Oncology Group; PS, performance status; EV, enfortumab vedotin; irAE, immune-related adverse event

The distribution of Eastern Cooperative Oncology Group performance status (ECOG PS) and metastatic sites was broadly comparable across groups. Treatment-related outcomes are shown in Table 2. Severe skin toxicity, peripheral neuropathy, and irAE were broadly comparable across groups. EV dose reduction was more frequent in the early-onset rash group than in the no-rash group after Holm adjustment (42.2% vs. 22.0%; Holm-adjusted *p* = 0.02), suggesting that early rash may reflect clinically meaningful treatment intolerance rather than a uniformly favorable toxicity phenotype. Among patients who developed skin toxicity, the median time to rash onset was 0.53 months (IQR, 0.27–1.40). The median onset time was 0.37 months (IQR, 0.23–0.54) in the early-onset rash group and 3.03 months (IQR, 1.65–5.58) in the late-onset rash group. Rash onset occurred predominantly within the first month after treatment initiation, whereas a smaller subset of patients developed delayed skin toxicity thereafter.

**Table 2:** Treatment-related outcomes according to skin-rash timing

Variable	No rash	Early-onset rash (< 1 month)	Late-onset rash ( > = 1 month)	*p* overall	*p* Early vs. No rash	*p* Late vs. No rash	*p* Late vs. Early
Skin toxicity Grade > = 3	0/87 (0.0%)	13/84 (15.5%)	8/39 (20.5%)	NA	NA	NA	0.61
Peripheral neuropathy	23/87 (26.4%)	20/84 (23.8%)	10/38 (26.3%)	0.91	1.00	1.00	1.00
irAE	16/85 (18.8%)	17/82 (20.7%)	9/38 (23.7%)	0.83	1.000	1.000	1.000
EV dose reduction during treatment	18/82 (22.0%)	35/83 (42.2%)	14/38 (36.8%)	**0.01**	**0.02**	0.24	0.69

Data are presented as number/total number (percentage). Pairwise p values were adjusted using the Holm method. Bold values indicate statistical significance (p < 0.05).

EV, enfortumab vedotin; irAE, immune-related adverse event

**Fig. 1:**
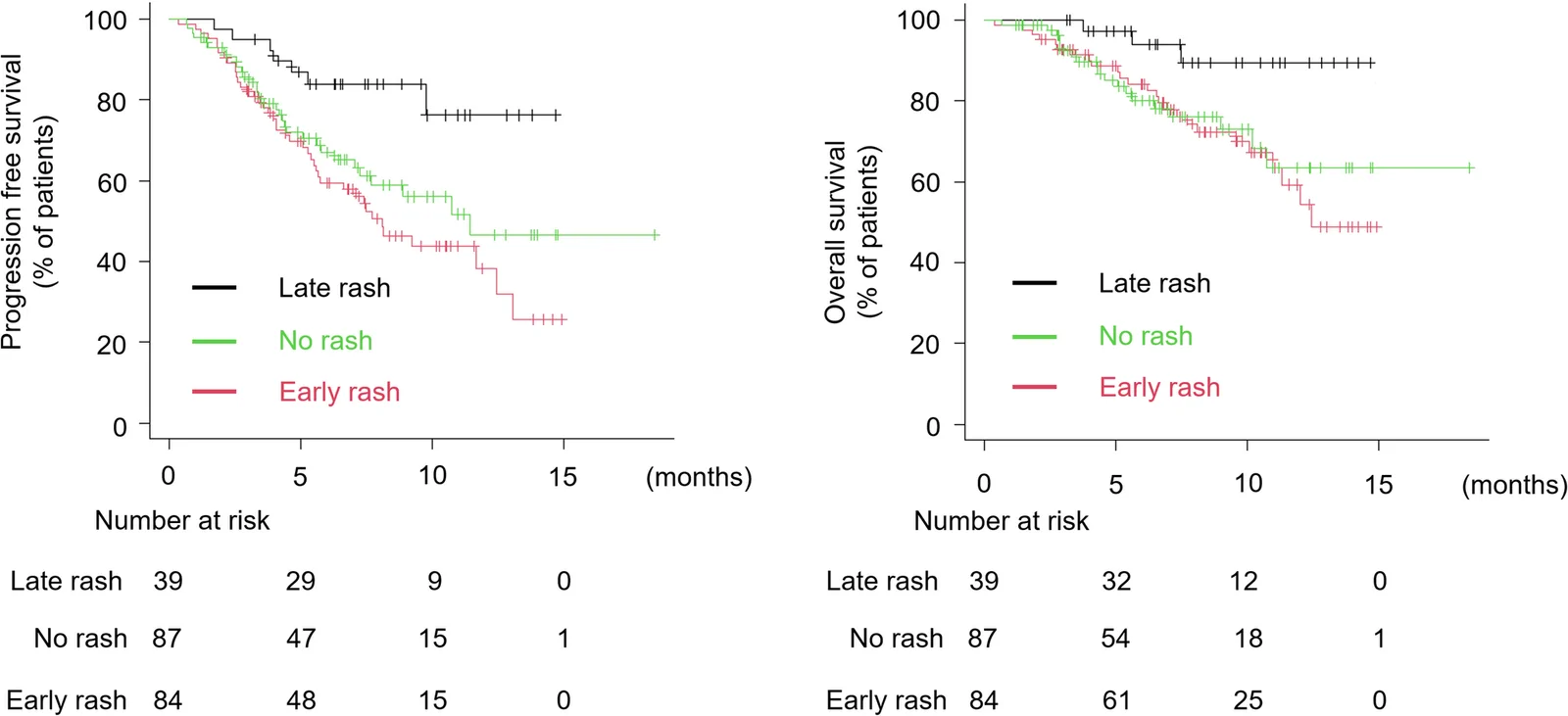
Kaplan-Meier curves according to skin-rash timing with log-rank analyses. **A** PFS according to no rash, early-onset rash (< 1 month), and late-onset rash (≥ 1 month). Global log-rank analysis demonstrated significant differences among the three groups (*p* = 0.006). Post-hoc pairwise analyses showed significantly prolonged PFS in the late-onset rash group compared with the early-onset rash group (Holm-adjusted *p* = 0.004) and the no-rash group (Holm-adjusted *p* = 0.04). No significant differences were observed between the no-rash and early-onset rash groups after Holm adjustment. **B** OS according to no rash, early-onset rash (< 1 month), and late-onset rash (≥ 1 month). Global log-rank analysis demonstrated significant differences among the three groups (*p* = 0.04). Post-hoc pairwise analyses showed significantly prolonged OS in the late-onset rash group compared with the early-onset rash group (Holm-adjusted *p* = 0.03) and the no-rash group (Holm-adjusted *p* = 0.049)

Survival outcomes are shown in Fig. 1. In the overall three-group Kaplan-Meier analysis, both PFS and OS differed significantly among the groups (PFS, *p* = 0.006; OS, *p* = 0.04). The late-onset rash group had the most favorable survival pattern. However, because patients with late-onset rash had to remain alive and under observation long enough to develop delayed rash, these unadjusted comparisons are vulnerable to immortal time bias. Post-hoc pairwise log-rank analyses demonstrated that late-onset rash was associated with significantly better PFS than both early-onset rash (Holm-adjusted *p* = 0.004) and no rash (Holm-adjusted *p* = 0.04). Late-onset rash was also associated with significantly better OS than both early-onset rash (Holm-adjusted *p* = 0.03) and no rash (Holm-adjusted *p* = 0.049). No significant differences in OS or PFS were observed between the no-rash and early-onset rash groups after Holm adjustment.

These findings indicate that late-onset rash was significantly associated with prolonged PFS and OS, whereas early-onset rash did not show a survival advantage over no rash. These results should be interpreted as hypothesis-generating rather than confirmatory.

Cox proportional hazards analyses are summarized in Table 3. In the OS analysis, late-onset rash was associated with significantly prolonged OS compared with the combined early-onset rash/no-rash group in univariate analysis (HR 0.25, 95% CI 0.08–0.81, *p* = 0.02) and remained significantly associated with prolonged OS after multivariable adjustment (HR 0.25, 95% CI 0.07–0.81, *p* = 0.02). ECOG PS ≥ 1 was significantly associated with worse OS (HR 3.17, 95% CI 1.75–5.76, *p* < 0.001), whereas liver metastasis showed a borderline association after multivariable adjustment (HR 2.05, 95% CI 0.97–4.34, *p* = 0.06).

**Table 3:** Univariate and multivariable Cox proportional hazards analyses for OS and PFS

Variable	Univariate HR	95% CI	*p* value	Multivariable HR	95% CI	*p* value
*A. Overall survival*						
Age continuous	1.01	0.98–1.04	0.52	0.99	0.96–1.03	0.91
Late-onset rash vs. Early-onset rash and No rash	0.25	0.08–0.81	**0.02**	0.25	0.07–0.81	**0.02**
ECOG PS > = 1	3.11	1.76–5.52	**< 0.001**	3.17	1.75–5.76	**< 0.001**
Liver metastasis	2.74	1.32–5.68	**0.007**	2.05	0.97–4.34	0.06
*B. Progression-free survival*						
Age continuous	0.99	0.98–1.02	0.96	0.99	0.97–1.02	0.54
Late-onset rash vs. Early-onset rash and No rash	0.33	0.15–0.72	**0.005**	0.34	0.15–0.74	**0.007**
ECOG PS > = 1	1.92	1.23–3.01	**0.004**	1.89	1.19–3.01	**0.007**
Liver metastasis	2.37	1.33–4.22	**0.004**	1.91	1.06–3.44	**0.03**

Variables with *p* < 0.05 in univariate analysis were entered into the multivariable model, and age was included as a clinically relevant covariate irrespective of its univariate p value. Bold values indicate statistical significance (p < 0.05)

In the PFS analysis, late-onset rash was associated with significantly prolonged PFS compared with the combined early-onset rash/no-rash group in both univariate (HR 0.33, 95% CI 0.15–0.72, *p* = 0.005) and multivariable analyses (HR 0.34, 95% CI 0.15–0.74, *p* = 0.007). ECOG PS ≥ 1 (HR 1.89, 95% CI 1.19–3.01, *p* = 0.007) and liver metastasis (HR 1.91, 95% CI 1.06–3.44, *p* = 0.03) were also associated with shorter PFS. Because rash timing was not modeled as a time-dependent exposure, these Cox results should not be interpreted as proving an independent causal effect of late-onset rash.

**Fig. 2:**
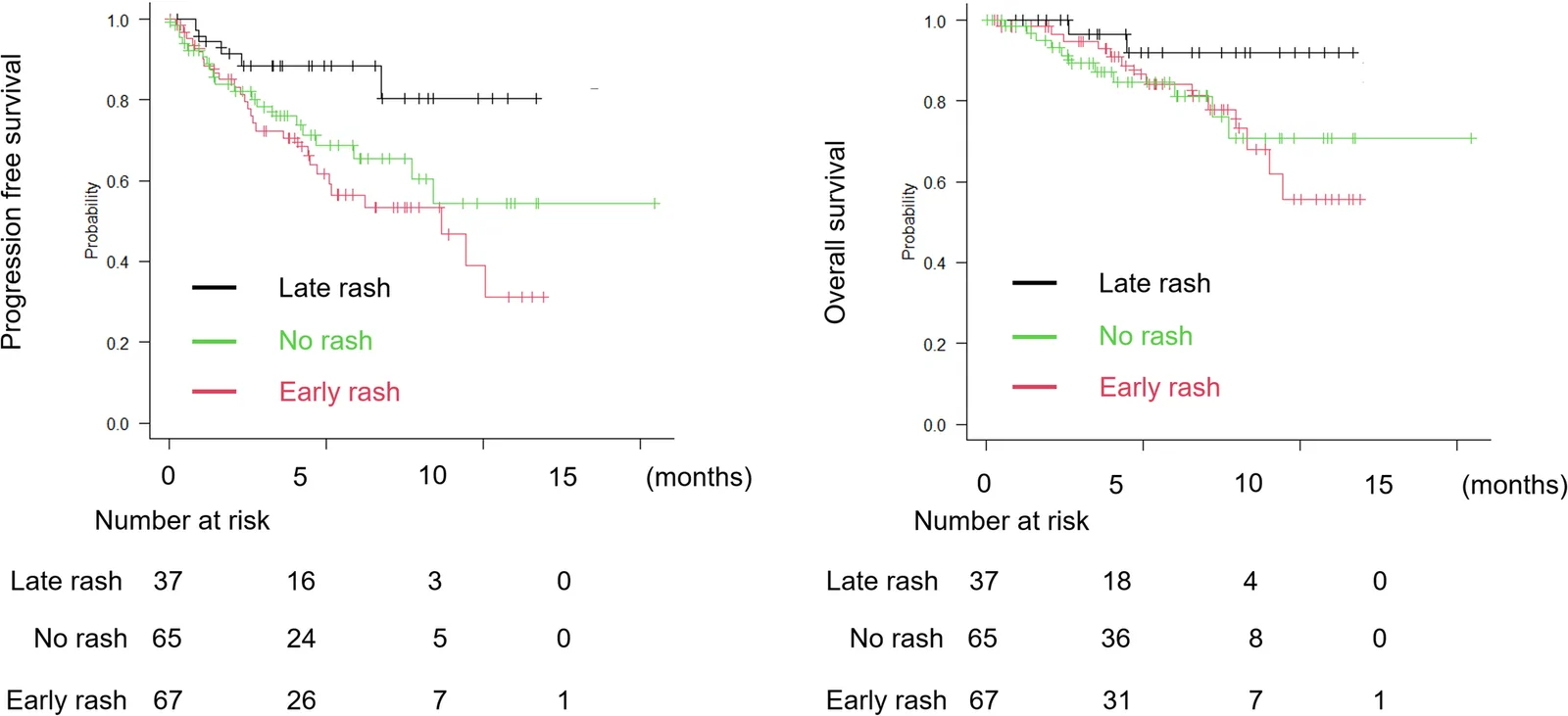
Kaplan-Meier curves in the 3-month landmark cohort according to skin-rash timing. Patients who experienced progression or death within 3 months after EV/Pem initiation or who had no follow-up beyond the 3-month landmark were excluded. PFS and OS were recalculated from the 3-month landmark, and the x-axis indicates months from the 3-month landmark. **A** PFS according to late-onset rash, no rash, and early-onset rash (< 1 month). The global log-rank test showed a significant difference among the three groups (*p* = 0.030). Pairwise log-rank analyses with Holm adjustment showed significantly prolonged PFS in the late-onset rash group compared with the early-onset rash group (Holm-adjusted *p* = 0.024), whereas the differences between late-onset rash and no rash and between early-onset rash and no rash were not significant (Holm-adjusted *p* = 0.132 and *p* = 0.269, respectively). **B** OS according to late-onset rash, no rash, and early-onset rash (< 1 month). The global log-rank test was not significant (*p* = 0.171). No pairwise comparison reached statistical significance after Holm adjustment (late-onset rash vs. early-onset rash, Holm-adjusted *p* = 0.20; late-onset rash vs. no rash, Holm-adjusted *p* = 0.20; early-onset rash vs. no rash, Holm-adjusted *p* = 0.87). Abbreviations: OS, overall survival; PFS, progression-free survival

In the 3-month landmark cohort, survival outcomes are shown in Fig. 2. After excluding patients who experienced progression or death within 3 months after EV/Pem initiation or who had no follow-up beyond the 3-month landmark, 169 patients were included in the landmark analysis: late-onset rash (*n* = 37), no rash (*n* = 65), and early-onset rash (*n* = 67). PFS and OS were recalculated from the 3-month landmark. In the PFS analysis, the global log-rank test showed a significant difference among the three groups (*p* = 0.030). The median PFS was not reached in the late-onset rash group, 8.67 months in the early-onset rash group, and not reached in the no-rash group. In pairwise log-rank analyses with Holm adjustment, PFS was significantly longer in the late-onset rash group than in the early-onset rash group (Holm-adjusted *p* = 0.024), whereas the differences between the late-onset rash and no-rash groups and between the early-onset rash and no-rash groups were not statistically significant (Holm-adjusted *p* = 0.132 and *p* = 0.269, respectively). For OS, the late-onset rash group showed a numerically favorable curve, but the global log-rank test was not significant (*p* = 0.171). No pairwise comparison reached statistical significance after Holm adjustment (late-onset rash vs. early-onset rash, Holm-adjusted *p* = 0.20; late-onset rash vs. no rash, Holm-adjusted *p* = 0.20; early-onset rash vs. no rash, Holm-adjusted *p* = 0.87).

## Discussion

This study suggests that skin toxicity during EV/Pem therapy could not be interpreted only as present or absent. Its timing appeared to define clinically distinct phenotypes: late-onset rash was associated with the most favorable survival pattern, with significantly prolonged PFS and OS, whereas early-onset rash was not significantly better than no rash. In the multivariable Cox analysis, late-onset rash remained significantly associated with prolonged PFS and OS compared with the non-late rash group. However, because late-onset rash necessarily occurs after a period of survival and continued observation, these findings should be viewed as exploratory associations that may be partly explained by immortal time bias. In the 3-month landmark analysis, the PFS difference between the late-onset and early-onset rash groups remained significant, whereas OS differences were not significant.

EV is a Nectin-4-directed antibody-drug conjugate that delivers MMAE into Nectin-4-expressing cells [2]. Nectin-4 expression in UC has been reported, supporting the biological relevance of Nectin-4-targeted treatment in UC [4]. Dermatologic events are recognized EV-related toxicities, and EV-associated skin toxicity is presumed to be related to MMAE delivery into Nectin-4-expressing normal epithelial structures, including epidermis and sweat glands [11–13]. Therefore, the timing of skin toxicity may plausibly reflect differences in epithelial tolerance to EV exposure. Because Nectin-4 is expressed not only in UC cells but also in normal epithelial structures, differences in epithelial responses to EV exposure may partly contribute to the distinct skin-toxicity phenotypes observed during EV/Pem therapy [11, 12].

Baseline KL-6 was higher in the early-onset rash group than in the late-onset rash group. KL-6 is a MUC1-derived epithelial marker used in the management of interstitial lung diseases, and prior studies have shown that KL-6 reflects epithelial injury and is useful for evaluating disease activity, prognosis, and treatment-related ILD [18–21]. These data do not prove that KL-6 is a skin-toxicity biomarker, but they support the interpretation that elevated baseline KL-6 may indicate a pre-existing epithelial-injury or epithelial-stress state. Importantly, KL-6 has not been validated as a predictive biomarker for dermatologic toxicity associated with EGFR inhibitors, immune checkpoint inhibitors, Stevens-Johnson syndrome, or antibody-drug conjugates. Therefore, the observed KL-6 difference should be interpreted cautiously, particularly considering the exploratory nature of the analysis and the multiple baseline comparisons performed.

MUC1 expression is not restricted to the lung. MUC1 is expressed in multiple epithelial tissues and has been reported in skin-related structures, including sweat glands, as well as in skin fibroblasts [22–25]. Thus, elevated KL-6 may reflect systemic epithelial stress rather than an isolated pulmonary phenomenon. Because EV targets epithelial Nectin-4-expressing structures [2, 11, 12], elevated baseline KL-6 may reflect baseline epithelial stress associated with susceptibility to early epithelial injury after EV exposure; however, this remains a hypothesis and requires validation. We therefore regard KL-6 as an exploratory signal rather than a clinically established biomarker of skin-toxicity susceptibility.

EV dose reduction was more frequent in the early-onset rash group than in the no-rash group. This supports the clinical relevance of early rash as a tolerability-related event. If skin toxicity were uniformly favorable, early-onset rash would be expected to show better survival than no rash; however, early-onset rash was not superior to no rash for either PFS or OS. This pattern suggests that early rash may be closer to treatment intolerance or epithelial fragility than to a beneficial efficacy marker. Although the frequency of grade ≥ 3 skin toxicity did not significantly differ between the early- and late-onset rash groups, rash severity and rash timing may represent distinct clinical phenomena. Severity reflects the maximum intensity of dermatologic toxicity, whereas timing may reflect when toxicity becomes clinically apparent in relation to treatment exposure and disease course.

Liver metastasis was less frequent in the late-onset rash group, but the difference did not reach statistical significance after Holm adjustment. Liver metastasis has been associated with systemic immunosuppression and reduced efficacy of immune checkpoint inhibitors, including mechanisms related to T-cell dysfunction or elimination [26, 27]. In addition, several real-world EV studies in metastatic UC have highlighted the importance of baseline clinical phenotype and metastatic burden for treatment outcomes [8–10, 13, 28–30]. Therefore, the lower frequency of liver metastasis in the late-onset rash group may have contributed to the favorable survival outcomes observed in this group. However, current evidence does not support a direct causal relationship between liver metastasis and skin-toxicity timing. Rather, liver metastasis may represent a confounding clinical factor associated with altered immune status or aggressive disease biology.

Taken together, these findings support a clinically testable model in which late-onset rash reflects preserved treatment exposure, favorable disease biology, and manageable delayed epithelial toxicity, whereas early-onset rash reflects baseline epithelial stress and treatment intolerance. This model is consistent with prior reports showing that EV-related outcomes and cutaneous toxicities in UC are influenced by multiple clinical factors [8–10, 13, 28–30], but it remains hypothesis-generating. The present data should not be interpreted as showing that delayed rash itself causally improves outcomes.

Several limitations should be emphasized. First, this was a retrospective exploratory analysis, and residual confounding could not be excluded. Second, immortal time bias is a major limitation of this study. Because late-onset rash was defined as skin toxicity occurring after 1 month of treatment, patients with early progression, death, or treatment discontinuation were structurally less likely to be classified into the late-onset rash group. This bias may substantially account for the favorable survival pattern observed in the late-onset rash group. A 3-month landmark analysis was performed to reduce this bias, and the PFS advantage of late-onset rash over early-onset rash remained significant in the landmark cohort, whereas OS differences were not significant. However, a 6-month sensitivity landmark analysis was not performed because the number of patients remaining at the 6-month landmark was limited, which could reduce statistical stability; time-dependent Cox models were also not performed. Therefore, the association between late-onset rash and survival should be interpreted cautiously and regarded as hypothesis-generating. Third, the biological relationship between KL-6 elevation, epithelial stress, and EV-related skin toxicity remains hypothetical and was not mechanistically validated.

## Conclusion

Skin toxicity during EV/Pem therapy appears to have timing-dependent prognostic implications. Late-onset rash showed favorable outcomes in conventional analyses, but this association may be influenced by immortal time bias. In the 3-month landmark analysis, late-onset rash remained associated with longer PFS than early-onset rash, whereas OS differences were not significant; therefore, these findings should be considered hypothesis-generating.

## Data Availability

Due to restrictions from the ethics review board, the data cannot be shared.

## Abbreviations

OS: Overall survival
PFS: Progression-free survival

## Declarations

### Reporting checklist

The authors have completed the STROBE reporting checklist.

## References

[1] Powles T, Valderrama BP, Gupta S et al (2024) Enfortumab vedotin and pembrolizumab in untreated advanced urothelial cancer. N Engl J Med 390:875–888 10.1056/NEJMoa2312117 38446675

[2] Challita-Eid PM, Satpayev D, Yang P et al (2016) Enfortumab vedotin antibody-drug conjugate targeting Nectin-4 is a highly potent therapeutic agent in multiple preclinical cancer models. Cancer Res 76:3003–3013 10.1158/0008-5472.CAN-15-1313 27013195

[3] Hashimoto M, Fukiage K, Minami T, De Velasco MA, Fujita K (2026) Treatment strategies for locally advanced and metastatic urothelial carcinoma based on molecular profiles: a review. Histol Histopathol 41:545–552. 10.14670/HH-18-972 40762043

[4] Hashimoto M, Fujita K, Tomiyama E, Fujimoto S, Toyoda S et al (2023) Immunohistochemical analysis of HER2, EGFR, and Nectin-4 expression in upper urinary tract urothelial carcinoma. Anticancer Res 43:167–174. 10.21873/anticanres.16146 36585180

[5] Tomiyama E, Fujita K, Hashimoto M, Adomi S, Kawashima A et al (2022) Comparison of molecular profiles of upper tract urothelial carcinoma vs. urinary bladder cancer in the era of targeted therapy: a narrative review. Transl Androl Urol 11:1747–1761. 10.21037/tau-22-457 PMC9827402 36632153

[6] Tomiyama E, Fujita K, Hashimoto M, Uemura H, Nonomura N (2024) Urinary markers for bladder cancer diagnosis: a review of current status and future challenges. Int J Urol. 10.1111/iju.15338 37968825

[7] Hashimoto M, Fujita K, Uemura H et al (2021) Higher neutrophil-to-lymphocyte ratio after the first cycle of the first-line chemotherapy is associated with poor cancer specific survival of upper urinary tract carcinoma patients. Transl Androl Urol 10:2838–2847. 10.21037/tau-21-185 PMC8350230 34430386

[8] Uchimoto T, Tsuchida S, Komura K, Fukuokaya W, Adachi T, Hirasawa Y, Hashimoto T, Yoshizawa A, Saruta M, Hashimoto M et al (2024) C-reactive protein-albumin ratio predicts objective response to enfortumab vedotin in metastatic urothelial carcinoma. Target Oncol 19:635–644. 10.1007/s11523-024-01068-7 38807017

[9] Hirasawa Y, Adachi T, Hashimoto T, Ohno Y et al (2024) Comparison of the efficacy of enfortumab vedotin between patients with metastatic urothelial carcinoma who were treated with avelumab or pembrolizumab: real-world data from a multi-institutional study in Japan. J Cancer Res Clin Oncol 150:182. 10.1007/s00432-024-05717-2 PMC11003883 38592548

[10] Hashimoto M, Fukiage K, Taniguchi K, Minami T, Fukuokaya W, Mori K et al (2025) Upper urinary tract urothelial carcinoma patients show better overall response rate than bladder cancer patients during enfortumab vedotin treatment following pembrolizumab. Transl Androl Urol 14:2279–2288. 10.21037/tau-2025-254 PMC12433179 40949456

[11] Lacouture ME, Sibaud V, Gerendash B et al (2022) Management of dermatologic events associated with the Nectin-4-directed antibody-drug conjugate enfortumab vedotin. Oncologist 27:e223–e232 10.1093/oncolo/oyac001 PMC8914492 35274723

[12] Egbeto IA, Vlachou E, Herrera DB, Russell S et al (2025) Clinical and histopathological characterization of enfortumab vedotin-associated cutaneous toxicities: a case series. JAAD Case Rep 57:114–121. 10.1016/j.jdcr.2024.12.019 PMC11928977 40124881

[13] Kita Y, Hara T, Kawahara T, Hashimoto K, Matsushita Y, Ito H et al (2025) Incidence, risk factors, and clinical implications of enfortumab vedotin-related cutaneous toxicity in urothelial carcinoma: a large-scale, real-world study in Japan. ESMO Real World Data Digit Oncol 7:100094. 10.1016/j.esmorw.2024.100094 PMC12836633 41647344

[14] Petrelli F, Borgonovo K, Cabiddu M, Lonati V, Barni S (2012) Relationship between skin rash and outcome in non-small-cell lung cancer patients treated with anti-EGFR tyrosine kinase inhibitors: a literature-based meta-analysis of 24 trials. Lung Cancer 78:8–15 10.1016/j.lungcan.2012.06.009 22795701

[15] Petrelli F, Borgonovo K, Barni S (2013) The predictive role of skin rash with cetuximab and panitumumab in colorectal cancer patients: a systematic review and meta-analysis of published trials. Oncology 85:167–175 10.1007/s11523-013-0257-x 23321777

[16] Rashdan H, Zhang S, Wan G et al (2024) Timing of cutaneous immune-related adverse events impacts survival in patients with cancer treated with immune-checkpoint inhibitors: a multi-institutional cohort study. J Am Acad Dermatol 90:1080–1083. 10.1016/j.jaad.2024.01.038 PMC11015977 38290617

[17] Kanda Y (2013) Investigation of the freely available easy-to-use software ‘EZR’ for medical statistics. Bone Marrow Transpl 48:452–458 10.1038/bmt.2012.244 PMC3590441 23208313

[18] Ishikawa N, Hattori N, Yokoyama A, Kohno N (2012) Utility of KL-6/MUC1 in the clinical management of interstitial lung diseases. Respir Investig 50:3–13 10.1016/j.resinv.2012.02.001 22554854

[19] Kondo T, Hattori N, Ishikawa N et al (2011) KL-6 concentration in pulmonary epithelial lining fluid is a useful prognostic indicator in patients with acute respiratory distress syndrome. Respir Res 12:32 10.1186/1465-9921-12-32 PMC3068089 21418654

[20] Kawase S, Hattori N, Ishikawa N et al (2011) Change in serum KL-6 level from baseline is useful for predicting life-threatening EGFR-TKIs induced interstitial lung disease. Respir Res 12:97 10.1186/1465-9921-12-97 PMC3160959 21791074

[21] Park HK, Lee Y, Kim Y et al (2023) Serum KL-6 levels predict the occurrence and severity of treatment-related interstitial lung disease in lung cancer. Sci Rep 13:18186 10.1038/s41598-023-45170-8 PMC10593856 37872370

[22] Brayman M, Thathiah A, Carson DD (2004) MUC1: a multifunctional cell surface component of reproductive tissue epithelia. Reprod Biol Endocrinol 2:4 10.1186/1477-7827-2-4 PMC320498 14711375

[23] Kato K, Lillehoj EP, Lu W, Kim KC (2017) MUC1: the first respiratory mucin with an anti-inflammatory function. J Clin Med 6:110 10.3390/jcm6120110 PMC5742799 29186029

[24] Kumar P, Ji J, Thirkill TL, Douglas GC (2014) MUC1 is expressed by human skin fibroblasts and plays a role in cell adhesion and migration. Biores Open Access 3:45–52. 10.1089/biores.2013.0045 PMC3995082 24804164

[25] Živná M, Kidd K, Přistoupilová A, Barešová V, DeFelice M, Blumenstiel B, Harden M, Conlon P, Lavin P, Connaughton DM, Hartmannová H, Hodaňová K, Stránecký V, Vrbacká A, Vyleťal P, Živný J, Votruba M, Sovová J, Hůlková H, Robins V, Perry R, Wenzel A, Beck BB, Seeman T, Viklický O, Rajnochová-Bloudíčková S, Papagregoriou G, Deltas CC, Alper SL, Greka A, Bleyer AJ, Kmoch S (2018) Noninvasive immunohistochemical diagnosis and novel MUC1 mutations causing autosomal dominant tubulointerstitial kidney disease. J Am Soc Nephrol 29:2418–2431. 10.1681/ASN.2018020180 PMC6115665 29967284

[26] Lee JC, Mehdizadeh S, Smith J, Young A, Mufazalov IA, Mowery CT, Daud A, Bluestone JA (2020) Regulatory T cell control of systemic immunity and immunotherapy response in liver metastasis. Sci Immunol 5:eaba0759 10.1126/sciimmunol.aba0759 PMC7755924 33008914

[27] Yu J, Green MD, Li S, Sun Y, Journey SN, Choi JE et al (2021) Liver metastasis restrains immunotherapy efficacy via macrophage-mediated T cell elimination. Nat Med 27:152–164 10.1038/s41591-020-1131-x PMC8095049 33398162

[28] Hashimoto M, Fukiage K, Taniguchi K, Minami T et al (2025) Cancer-induced pain is associated with poor overall survival of urothelial carcinoma patients treated with enfortumab vedotin. Vivo 39:1533–1539. 10.21873/invivo.13953 PMC12041981 40295006

[29] Nishio K, Hirosuna K, Uchimoto T, Niigawa H et al (2025) Real-world outcomes of enfortumab vedotin in elderly patients with metastatic urothelial carcinoma. Anticancer Res 45:4575–4583. 10.21873/anticanres.17803 41006037

[30] Taniguchi K, Hashimoto M, Nakayama T, Fujimoto S, Toyoda S et al (2026) A case of upper tract urothelial carcinoma with neuroendocrine differentiation successfully treated with enfortumab vedotin and pembrolizumab. IJU Case Rep. 10.1002/iju5.70149 PMC12920264 41726849

